# Deep-sea mining and its risks for social-ecological systems: Insights from simulation-based analyses

**DOI:** 10.1371/journal.pone.0320888

**Published:** 2025-03-28

**Authors:** Lubna Alam, Kumara Perumal Pradhoshini, Raphaelle A. Flint, U. Rashid Sumaila

**Affiliations:** 1 Fisheries Economics Research Unit, Institute for the Oceans and Fisheries, The University of British Columbia, Vancouver, BC, Canada; 2 Institute for Environment and Development (LESTARI), The National University of Malaysia (UKM), Bangi, Selangor, Malaysia; 3 Independent Researcher, Chennai, Tamil Nadu, India; 4 Dona Bertarelli Philanthropy, Ledunfly SA, Nyon, Switzerland; 5 School of Public Policy and Global Affairs, The University of British Columbia, Vancouver, BC, Canada; 6 Department of Agricultural Economics and Rural Development, University of Pretoria, Pretoria, South Africa; Maria Curie-Sklodowska University: Uniwersytet Marii Curie-Sklodowskiej, POLAND

## Abstract

The pros and cons of deep-sea mining (DSM) is currently hotly debated. Here, we assess the environmental, economic, and social risks of DSM by comparing scenarios with and without DSM involvement. The “Without” scenario relies solely on land-based mining and circular economy solutions, while the “With” scenario incorporates DSM alongside circular strategies, highlighting the dangers of heavy DSM dependence. Through literature review and expert interviews, our study identifies key risk indicators across environmental, economic, and social dimensions, forming a comprehensive assessment framework. Through the application of qualitative data and fuzzy cognitive mapping, the analysis reveals that environmental factors are the most influential (centrality: 1.46), followed by social (1.32) and economic (1.0) factors. In the “With DSM” scenario, all indicators show increased risks, with environmental factors, particularly “coastal state vulnerability,” experiencing a 13% rise. Social risks, including “violation of law,” “participatory rights,” “lack of effective control,” and “degraded reputation,” increase by 8–11%, while economic risks, such as “contractual violations,” “lack of special provision,” “knowledge gap on economic assistance fund” and disputes among “multiple stakeholders,” see an 11% uptick. Our results suggest that the risks DSM poses to deep-sea marine ecosystems are likely too significant to justify its pursuit and advocates for circular economy solutions as viable alternatives to mitigate environmental, social, and economic risks. We recommend that policies should promote circular practices through resource recovery incentives.

## Introduction

Deep-sea environments are among the most fragile and poorly understood on Earth, and they are home to distinctive and inadequately investigated species that are crucial for the stability and health of marine ecosystems [1]. Consequently, while deep-sea mining (DSM) could contribute to the supply of critical metals for industries such as electronics, renewable energy, and automotive manufacturing [2], the anticipated environmental costs are significant [3,4]. A study on DSM of cobalt-rich crusts on the Rio Grande Rise highlights disturbances such as substrate removal, increased suspended particulate matter, crushing by tailings, and toxicity from released metals [5]. These impacts are expected to alter the ecosystem and its functioning for decades to centuries. Climate models predict increased biomass for bigeye, skipjack, and yellowfin tuna in the Clarion-Clipperton Zone (CCZ) due to climate change. Thus, interactions between mining, fish populations, and climate change remain complex, however, these shifts suggest potential for greater environmental and economic conflicts [6]. As a whole, DSM is likely to significantly affect the delicate ecosystems and the services they provide, including provisioning, supporting, regulating, and cultural services [7,8].

Economically, DSM entails significant expenses and risks associated with technological advancement, operational logistics, and regulatory adherence in sometimes remote and challenging settings [9]. The development of specialized mining machinery and deep-sea technologies requires substantial capital investment, accompanied by considerable risks of technical malfunction owing to the severe pressure and corrosive environment of the deep ocean [10]. Operational logistics exacerbate these issues, necessitating sophisticated support vessels, comprehensive maintenance, and proficient people, all of which escalate expenses [11]. Furthermore, adherence to international and national regulatory frameworks may incur additional expenses and delays, especially as DSM laws continue to develop [12]. In the CCZ, pilot projects encountered persistent delays attributable to technical and regulatory challenges [13,14]. These considerations collectively classify DSM as a high-risk economic endeavor, with profitability dependent on assumed increasing global demand for essential minerals and consistent regulatory frameworks. Moreover, the financial viability of DSM projects is closely tied to global market conditions, technological advancements, and regulatory frameworks that are still evolving [15].

Socially, DSM raises questions regarding equity, Indigenous rights, and the involvement of local communities in decision-making processes [16]. Scholars argue that the distribution of benefits and risks from DSM activities often mirrors existing global inequalities, disproportionately impacting local coastal communities and Indigenous populations who may derive their livelihoods from the marine environment [17]. These groups frequently face marginalization in decision-making processes despite being primary stakeholders affected by resource extraction activities [18]. The failure to involve these communities meaningfully in consultations and benefit-sharing arrangements has raised concerns about violations of their social, cultural, and economic rights [19]. Equally problematic is the limited transparency in DSM decision-making, which often excludes local voices and limits opportunities for affected communities to influence policies or practices [20]. To address these concerns, experts emphasize the need for inclusive governance structures that recognize the rights and knowledge of local communities which are essential to balance the economic potential of DSM with its social responsibilities [21].

Since 2017, the International Seabed Authority (ISA) has been developing a Mining Code to regulate seabed mineral extraction beyond national jurisdictions. Environmental regulations are playing an increasingly significant role in these negotiations, influencing key aspects such as spatial planning strategies, contract approval procedures and environmental impact assessment guidelines [22]. Recent discussions at the ISA Council Meeting in March 2024 highlighted questions regarding the integration of environmental issues into DSM regulations and contractual frameworks [23]. The debates emphasized the necessity of exploring measures to address environmental externalities, recognizing that environmental considerations are essential for sustainable development rather than merely regulatory obligations. However, the extent to which these measures will have a meaningful impact on regulatory outcomes remains uncertain.

The concept of the circular economy has gained prominence as a sustainable approach, offering alternatives to address the broader environmental challenges, including those posed by DSM [15,24]. Continuous advancements in material efficiency and resource substitution have already decreased the amount of minerals needed for battery technology. Lithium-iron-phosphate batteries are an example of such batteries because they do not require nickel or cobalt, which are now in high demand but this demand is likely to decline with time [15]. While this raises doubts about the immediate necessity of DSM, it is recognized that further research is required to substantiate these claims. The circular economy paradigm increases resource efficiency through recycle, reuse, and sustainable management practices to minimize waste and reduce environmental footprints [25,26]. Circular economy approaches also influence mineral demand and supply by reducing the total annual demand for minerals because (i) they prolong the lifespans of infrastructure and services, allowing extracted minerals to be used in society for a longer period of time; and (ii) they control the timing and quantity of material recovery, allowing these materials to re-enter the production process as replacements for primary raw materials [24]. As a result, urban mining, which recovers metals from discarded electronic and industrial waste [27,28] can act as a more sustainable and resource-efficient alternative to DSM. Advances in recycling technologies have made it possible to extract valuable metals such as cobalt, nickel, and rare earth elements from e-waste [29,30]. Therefore, integrating circular economy principles into metal supply strategies has the potential to eliminate the need for DSM and its related hazards, boosting environmental sustainability and socioeconomic resilience.

Previous research has largely focused on isolated aspects of DSM, such as environmental, social, or economic risks, or explored the theoretical benefits of circular economy strategies. However, these studies often lack a comprehensive approach that integrates these perspectives to assess policy implications, particularly within the context of DSM. The present study addresses these gaps by evaluating the potential impacts of DSM on social-ecological systems while contrasting them with the benefits offered by circular economy strategies. Through the adoption of an integrated framework that encompasses environmental, socio-economic, and governance dimensions, the research aims to provide valuable insights for policy and management decisions on whether to pursue or forgo DSM activities. Simas et al. [24] asserts that “the future is circular,” and highlight the potential of reducing the demand for seven important raw minerals that can be supplied by DSM by 58% between now and 2050 through the adoption of new technologies, circular economy models, demand reduction techniques, and improved recycling. An Environmental Justice Foundation report also highlighted how DSM could impede the shift to a circular economy by redirecting investments away from sustainable solutions [15]. Building on the assumption that future metal demands could be reduced by up to 58% through “circular solutions” including circular economy practices, new technologies, and recycling, the current study offers a strategic assessment to inform sustainable resource management and promote alternative solutions that minimize ecological and social risks.

## Materials and methods

### Identifying indicators

The planning and execution of this research involved a first phase of literature review aimed at finding indicators to determine the risks associated with DSM activities based on the current state of the DSM industry. The search focused on peer-reviewed articles, reports and online databases available on Google, which yielded approximately 70 articles, forming the bases for the analysis. The indicators were carefully chosen to capture both direct and indirect risks associated with DSM activities, encompassing immediate operational impacts as well as long-term consequences for various stakeholders and ecosystems. Those that were measurable or assessed with standard qualitative and quantitative methods in literature were considered, to ensure data validity. On the other hand, indicators from any unsupported evidence of literature and those with significant overlap or redundancy with other selected indicators were excluded.

The indicators were categorized into three major classes: i) environmental indicators: assessing the likely impacts of DSM on the environment; ii) economic indicators: evaluating cost factors in mining, including capital and operational expenditures, as well as environmental costs; and iii) social indicators: focusing on social aspects, including potential and conflicting interests of Indigenous communities. Environmental indicators were selected based on their ability to measure both immediate ecosystem disruption and long-term environmental consequences. Through this, we identified 8 environmental indicators, i.e., biodiversity risk; vulnerability to coastal states; pollution; troublesome sea life; knowledge gaps; greenwashing argument; ambiguous guidelines; and habitat risk. Detailed descriptions of each of these are provided in S1 Table in S1 File. Economic indicators were chosen to reflect both micro and macro-economic implications, ranging from direct operational costs and profitability risks to broader impacts on existing industries and national economies. We identified 12 economic indicators consisting of the following: private profit over public good; risk on commercial fisheries; ecological cost; technological cost; loss and profitability risks; tourism; royalty payment regimes; knowledge gap on EAF; terrestrial mining vs DSM; multiple shareholders; lack of special provision; and contractual violations. We provide detailed descriptions of each of these indicators in S2 Table in S1 File. Social indicators were selected to capture the complex interplay between DSM operations and societal well-being. Total 8 social indicators were identified, including: political implications; social stigma; food security and safety; lack of effective control; violation of the law; degraded reputation; livelihood and employment loss; and participatory rights. Detailed descriptions of each of these are provided in S3 Table in S1 File.

S4 diagram in S1 File illustrates the interconnected risks associated with DSM across environmental, economic, and social dimensions, highlighting the importance of a comprehensive assessment framework. Environmental indicators are crucial as they address the potential for irreversible damage to deep-sea ecosystems, emphasizing the need for conservation measures and adherence to global sustainability goals. Economic indicators are essential for evaluating the feasibility of DSM operations, particularly considering their implications for existing maritime industries and the economies of developing maritime nations. Equally important are social indicators, which provide insights into the broader social implications of DSM, including community well-being, governance, and employment. This integrated framework offers a structured approach to understanding and evaluating the multifaceted risks associated with DSM, enabling more informed and sustainable decision-making processes.

### Data collection

After identifying the risk indicators, the questionnaires were developed for obtaining the responses from experts in the field. The questionnaire consisted of 2 sub questions for each individual major question: i) what is the likelihood that the proposed risk burden from DSM will affect the social-ecological systems; and ii) how significant would the resulting impact be? Interviews were conducted with nine experts, including representatives from NGOs, academia, and a DSM mining company, to capture perceptions of DSM risks. Detailed information is provided in (Table 1). Experts were asked to provide their views on the 41 identified questions, encompassing environmental, economic, and social risk indicators. The responses obtained were transcribed and consolidated into different categories using Microsoft excel.

**Table 1:** Expert profile for risk indicators interview.

Category	Distribution of respondents	Percentage (%)
Highest Education Level		
	Bachelor	0
	Master’s Degree	56
	Ph.D.	44
Field of Work		
	Environmental Sustainability	22
	Environmental Policy & Governance	22
	Law & Regulatory Affairs	33
	Economics & Resource Management	22
Organization		
	University Researchers & Professors	44
	Industry Experts	11
	Policy Advisors & Government Officials	0
	NGO Representatives & Advocacy Specialists	44

We collected the indicators for this study through an extensive literature review. Expert opinions were sought solely to verify the relevance of the data, and their involvement was limited to providing feedback on pre-existing information. No personal or sensitive data were collected. Due to the non-sensitive nature of the data, written consent was not required. However, verbal consent was documented through meeting notes to ensure transparency and compliance with ethical standards.

### Construction of Fuzzy Cognitive Map (FCM)

This study employs a Fuzzy Cognitive Map (FCM) to assess the perceived influence of DSM on marine ecosystem services. FCMs, originally proposed by Kosko [31], are computational models that combine fuzzy logic, neural networks, and cognitive mapping techniques [31,32]. These models are composed of several components and the causal connections between them, which together create a neural network [32,33]. Every component is given an activation value (AV), i.e., the degree of impact it has on the network of between 0 and 1, and each relationship is allocated a weight that represents the degree of causality, ranging from -1 to 1 (where 1 and -1 signify full positive and negative causality, respectively). Fuzzy logic is employed to transform both quantitative and qualitative data into a common format, which is then used to determine the AVs and weights [34]. In the present study, the FCM framework was applied to a hypothetical DSM project, integrating various risk indicators and expert opinions. Qualitative data from expert interviews have been used to calculate total risks multiplying the likelihood by the severity using the following equation,


RiskScore=Likelihood×Impact (1)

Where:

Likelihood =  the probability of the risk event occurring (from 1 to 5).

Impact =  the severity of the consequences if the event occurs (from 1 to 5).

The risk score is used to determine the weight in the FCM, where the weight represents the degree of causality or influence between components. For example, a risk score of 25 corresponds to a weight of + 1, indicating a strong positive causality between the two components involved. This means that when both the likelihood of occurrence and the impact are high, the relationship between the components is considered fully positive, signifying that the risk has a significant influence on the network.

The second phase of the literature review is aimed at discovering the most significant indicators, which are linked to circular solutions (CS) encompassing circular economy, new technology and recycling. The phase involved reviewing 21 published sources (S4 Table in S1 File) to determine which keywords were most frequently associated with impacts on circular solutions. Each publication was categorized by the first author’s name and given a score of 1 when an indicator was mentioned and 0 when it was not. The assessment involved adding all of the scores and determining the average score per indicator across all variables using the method described by Cordova-Pozo and Rouwette [35]. The Indicator Score was calculated using the formula below,


$$IndicatorScore=\frac{Sumofscoresforindicator}{21}$$ (2)

**Fig 1:**
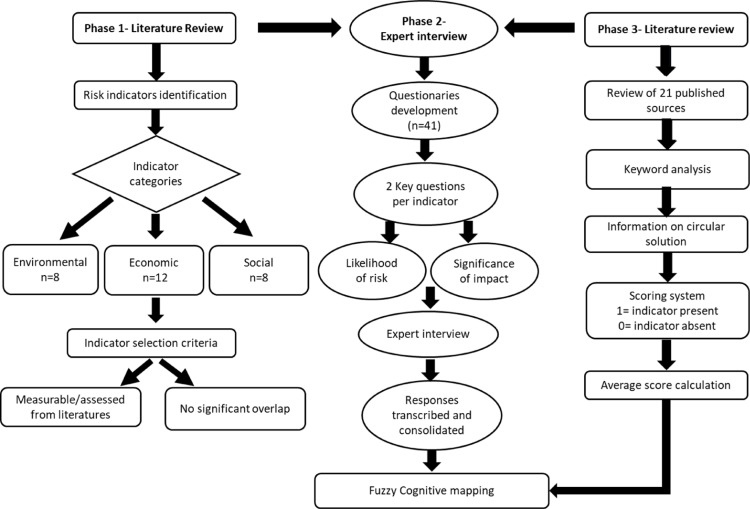
Framework for risk assessment of deep-sea mining using fuzzy cognitive mapping.

The calculated Indicator Scores were subsequently used to determine the weights in the FCM, with each weight being directly proportional to the Indicator Score. For instance, an indicator with a score of 0.81 would have a corresponding weight of 0.81, reflecting the strength of the causality between components. These weights were then input into the “Mental Modeler” software to construct the FCM, which modeled the relationships between components based on their calculated weights, enabling a comprehensive analysis of the factors influencing circular solutions. The overall risk assessment methodological flowchart is presented in (Fig 1).

The figure depicts the stepwise framework for assessing risks associated with DSM. The process involves three phases. Phase 1 begins with a literature review to identify risk indicators, categorized into environmental, economic, and social aspects. Phase 2 entails expert interviews to evaluate the likelihood of risks and the significance of impacts through a questionnaire. The responses are transcribed and consolidated for analysis. Phase 3 incorporates a review of published sources to analyze the impact of indicators on circular solutions using keyword analysis, scoring systems, and average score calculations. The outputs from all phases feed into the development of the FCM for a comprehensive risk assessment.

To quantify the FCM, we calculated the following metrics [36,37]:

- Number of Concepts (N): The total number of unique variables or nodes in the FCM;
- Number of Connections (C): The total number of directed edges or connections between the concepts;
- Connections per Concept (C/N): The average number of connections per concept;
- Density (D): An index of connectivity, calculated by dividing the number of connections by the maximum number of connections possible between N variables;
- Driver Variables: Positive outdegree and zero indegree, indicating they drive changes in the system;
- Receiver Variables: Positive indegree and zero outdegree, indicating they are influenced by other variables; and
- Ordinary Variables: Non-zero indegree and outdegree, indicating they both influence and are influenced by other variables.

**Fig 2:**
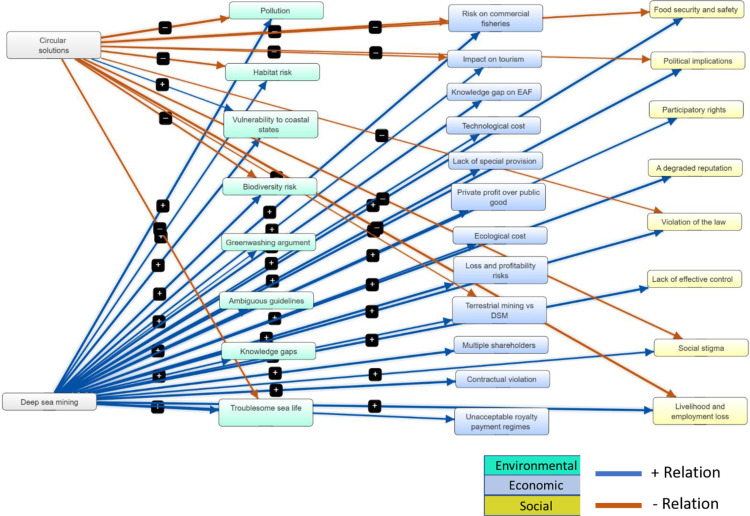
Fuzzy cognitive model of deep-sea mining and circular solutions for future metal supply. The constructed Fuzzy Cognitive Map (FCM) represents a complex system with 30 key elements linked by 41 interconnections. It features 2 driver components, “deep-sea mining” and “circular solutions”, which significantly influences many other elements. “Deep-sea mining” emerges as the element with the highest centrality, highlighting its pivotal role and extensive connectivity within the system. Its extensive connectivity highlights the widespread social-ecological implications of DSM, emphasizing the importance of carefully evaluating mining practices and exploring circular economy strategies to mitigate negative consequences.

The FCM model developed in this study (Fig 2) represents a system with 30 key elements interconnected through 41 relationships (Table 2). The density of the FCM 0.05, which is calculated as the ratio of the actual number of connections to the total possible connections in the map. On average, each component has approximately 1.36 connections. This metric provides insights into the average connectivity within the map. It has 2 driver components and 28 receiver components, indicating a dynamic system with multiple feedback loops and a moderate level of complexity (Table 3).

**Table 2:** General Fuzzy Cognitive Map (FCM) statistics.

FCM properties	Value
Total Components	30
Total connections	41
Density	0.05
Connections per component	1.36
Number of driver components	2
Number of receiver components	28
Number of ordinary components	0
Complexity score	14

**Table 3:** Concept categorization and network metrics in the Fuzzy Cognitive Map (FCM).

Category	Component	Indegree	Outdegree	Centrality	Type
Economic	Private profit over public good	0.9	0	0.9	Receiver
	Risk on commercial fisheries	1.81	0	1.81	Receiver
	Ecological cost	0.6	0	0.6	Receiver
	Technological cost	0.6	0	0.6	Receiver
	Loss and profitability risks	0.85	0	0.85	Receiver
	Impact on tourism	1.23	0	1.23	Receiver
	Unacceptable royalty payment regimes	0.8		0.8	Receiver
	Knowledge gap on EAF	1	0	1	Receiver
	Terrestrial mining vs DSM	1.18	0	1.18	Receiver
	Multiple shareholders	1	0	1	Receiver
	Lack of special provision	1	0	1	Receiver
	Contractual violation	1	0	1	Receiver
Environmental	Biodiversity risk	1.58	0	1.58	Receiver
	Vulnerability to coastal states	1.38	0	1.38	Receiver
	Pollution	1.85	0	1.85	Receiver
	Troublesome sea life	1.56	0	1.56	Receiver
	Knowledge gaps	1.8	0	1.8	Receiver
	Greenwashing argument	0.48	0	0.48	Receiver
	Ambiguous guidelines	1.64	0	1.64	Receiver
	Habitat risk	1.37	0	1.37	Receiver
Social	Political implications	1.38	0	1.38	Receiver
	Social stigma	1.51	0	1.51	Receiver
	Food security and safety	1.81	0	1.81	Receiver
	Lack of effective control	1	0	1	Receiver
	Violation of the law	1.29	0	1.29	Receiver
	A degraded reputation	1	0	1	Receiver
	Livelihood and employment loss	1.9	0	1.9	Receiver
	Participatory rights	0.64		0.64	Receiver
Metal sources	Circular solutions	0	7.90	7.90	Driver
	Deep-sea mining	0	24.28	24.28	Driver

In the context of FCM, the outdegree, indegree, and centrality of variables are key metrics used to evaluate the role and influence of each concept within the system. These measures help categorize variables as driver, receiver, or ordinary based on their connectivity and the direction of influence within the network. The outdegree of a variable refers to the cumulative strength of the connections that exit from that variable. It quantifies the influence that the variable exerts on other variables in the system. The outdegree is calculated as the sum of the weights of all outgoing edges from a variable. The indegree of a variable measures the cumulative strength of the connections entering that variable. It indicates how much a variable is influenced by other variables in the system. A higher outdegree indicates that the variable has a greater influence on other variables in the system. A higher indegree suggests that the variable is more influenced by other components in the system [36,37].

The centrality of a variable is the total sum of its outdegree and indegree, indicating its overall importance within the system. A variable with high centrality is more influential, either as a driver of change (outdegree) or as an important receiver of influence (indegree) and it is measured by the formula below [38],


CentralityofX=OutdegreeofX+IndegreeofX (3)

In this analysis “deep-sea mining” has the highest centrality (24.28), indicating its significant influence and connectivity within the map (Table 3).

In the current study, the FCM approach was preferred over methods like the Analytic Hierarchy Process (AHP) due to its ability to model complex, dynamic systems with interdependencies and feedback loops [39]. Unlike AHP, which is static and focuses on hierarchical comparisons [40], FCM captures the non-linear interactions between ecological, economic, and technological factors [41]. FCM also integrates both qualitative and quantitative data, accommodates uncertainty, and offers a deeper understanding of causal relationships [42], making it more adaptable for evaluating the interconnected impacts of DSM on marine ecosystem services.

To address uncertainties inherent in the FCM approach, we conducted scenario analysis to examine the robustness of model outcomes under different assumptions. By systematically varying key connection weights and testing alternative configurations of the model, we assessed the extent to which uncertainties in expert-defined relationships influence system behavior. The results demonstrated that while some concepts exhibited sensitivity to specific changes, the overall system dynamics remained consistent across scenarios, reinforcing the model’s reliability.

### The “With” and “Without” DSM analyses

Running the Mental Modeler in FCM involves assigning values ranging between -1 and + 1 to represent the influence or impact of one element on another within a model. Assigning different values in the model is used to simulate and predict the outcomes of different scenarios. When setting up scenarios in FCM, a weight of 0.1 might be assigned to a variable to reflect that this variable has a minor or limited influence over other variables within the FCM model. To the best of our knowledge, there is no existing research that establishes the current state and values of the social, environmental, and economic concepts related to DSM that could be incorporated into the creation of the FCM needed for our analysis. Therefore, following the concept of Kokkinos et al. [32] and Hatziioannou & Kokkinos [43], we consider the steady state of the FCM model as the status quo scenario.

#### The “Without” DSM analysis.

Here, mineral supplies are assumed to be met fully from land-based mining and circular economy solutions with no supplies derived from DSM. The status quo scenario described earlier is assumed to represent the “Without” DSM situation.

#### The “With” DSM analysis.

Here, high quantities of mineral supplies are derived from both DSM and CS (leveled as + 1 in Mental Modeler). This scenario aims to highlight the potential risks and challenges associated with heavy reliance on DSM for meeting metal demands.

We found considerable percentage changes in the AVs with DSM relative to the “Without” DSM scenario. The % change in AV functions as a relative measure, rather than an absolute value, where higher percentage changes indicate a more significant influence on the specific component being considered. To qualitatively describe the magnitude of these relative changes, in AV, the following categorization was used: “significant” for differences greater than 1.0%; “slight” or “minor” significance for differences between 1 and 3.0%; “moderate” significance for differences ranging 3.1% to 7.0%; and “large” or “greatly” significance for differences exceeding 7.1% [44].

## Results

Among the economic factors considered in this study, “risk on commercial fisheries” (1.81) and “impact on tourism” (1.23) are key drivers due to their higher centrality values indicating direct impact on economic performance, suggesting the need to address these risks for stability. Economic indicators with moderate centrality value, such as, “private profit over public good” (0.9) reflect the tension between profit-driven decisions and social welfare, requiring balanced policies, while lower centrality indicators like “ecological cost” (0.6) indicate overlooked long-term consequences. Similarly, environmental factors with higher centrality values like “pollution” (1.85) and “knowledge gaps” (1.8) dominate due to their critical role in environmental degradation and inefficiencies, showing that addressing them could produce cascading benefits. Social factors such as “livelihood and employment loss” (1.9) and “food security and safety” (1.81) are the most critical, reflecting their immediate impact on community well-being, while moderate factors like “social stigma” (1.51) and “political implications” (1.38) emphasize the importance of social cohesion and governance. Low-centrality factors like “participatory rights” (0.64), though less significant, are enabling components that indirectly enhance systemic interventions. The data gathered and analysed clearly indicate that, on average, environmental variables, with an average centrality score of 1.46, have a greater influence and relevance within the FCM compared to social components with an average centrality score of 1.32, and then economic factors with an average centrality score of 1.0. High environmental centrality indicates that the environmental risks linked to DSM such as biodiversity loss, pollution, habitat disturbance, and vulnerability to coastal states, are significant factors that affect the overall FCM system dynamics.

### The effects of deep-sea mining

**Fig 3:**
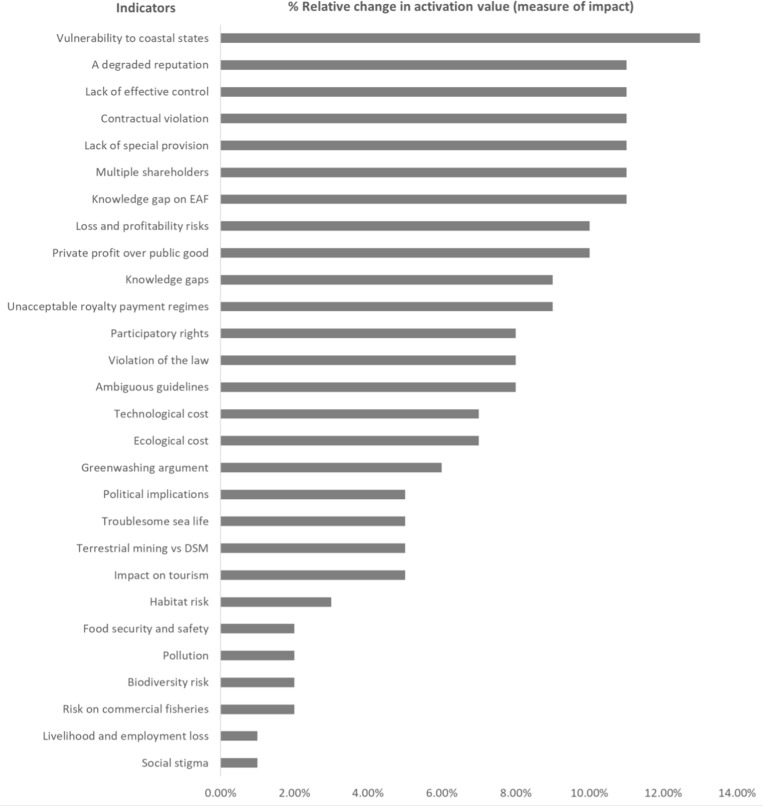
Relative changes in DSM impacts across environmental, social and economic indicators. This figure illustrates the percentage changes from a steady state (our assumed “Without” DSM scenario) in activation values - a measure of the impacts of DSM across various indicators. The indicators, listed on the y-axis, reflect different dimensions of environmental, social, and economic impacts. The x-axis represents the percentage change in activation values for each indicator. Activation values are critical in this context as they help quantify the magnitude of DSM’s impact, providing a clearer picture of how various elements of the system are affected. Percentage changes in these values highlight key insights, such as the increased vulnerability of coastal states and reputational risks, which emerge as the most significantly impacted indicators.

The findings of the “With” DSM analysis for all the studied indicators are presented in (Fig 3). The immediate observation from the figure is that DSM increases the risks associated with all indicators. The highest likely risk imposed by DSM is on coastal states as measured by the “vulnerability to coastal states” indicator at an increase of 13% relative to the “Without” DSM. for example, mining activities in the CCZ pose a significant threat to Small Island Developing States in the region, compounding the existing challenges these nations face due to the continuous rise in sea levels [45]. These dual threats, environmental degradation from DSM and the long-term impacts of climate change, create a precarious situation for these small island countries, with profound implications for their ecosystems, economies, and societies.

Social indicators that register high increases in risks are “violation of law,” “participatory rights,” “lack of effective control,” and “degraded reputation,” with relative increases of 8% to 11%. The “violation of law” as a social risk in DSM highlights legal concerns stemming from insufficient environmental assessments. For instance, in Norway, advancing DSM without robust scientific evidence could breach national and international laws [46]. Such actions pose a risk of undermining environmental protections and may provoke legal challenges, emphasizing the necessity of adhering to regulations in order to preserve regulatory integrity and public trust [47]. “Participatory rights” in the context of DSM refer to the procedural rights of stakeholders, including Indigenous peoples, local communities, and the general public, to actively engage in decision-making processes. Currently, the legal framework governing DSM often falls short of fulfilling state obligations to ensure meaningful stakeholder involvement. Incorporating indigenous peoples into DSM management is not just a procedural requirement but a fundamental aspect of their comprehensive, tradition-based connection to the environment, as acknowledged by international initiatives and the regulations of Pacific States [48,49]. Similarly, “lack of effective control” in DSM refers to the insufficient oversight and authority of sponsoring states over the companies they endorse for mining activities. Furthermore, the ISA has not clearly defined what constitutes “effective control,” leading to ambiguities. For example, in the case of TOML, Tonga’s ability to fulfill its regulatory responsibilities is uncertain, highlighting the critical need for clearer definitions and stronger governance mechanisms [50]. On the other hand, “degraded reputation” highlights the damage to a state’s image due to controversial decisions. In particular, Norway’s decision to open its seabed for commercial DSM, despite warnings from scientists and environmental experts, has drawn criticism potentially eroding trust and credibility in its environmental commitments [51].

We also see large economic risks, especially in areas such as “contractual violation,” “lack of special provision,” “multiple shareholders,” “knowledge gap on EAF,” “loss and profitability risks”, “private profit over public good” and “unacceptable royalty payment regimes”. “Contractual violation” in DSM refers to the potential breach of agreements between companies and regulatory bodies, such as the ISA. For example, if TOML, a subsidiary sponsored by Tonga, fails to meet its contractual obligations, the sponsoring state (Tonga) could face significant financial liabilities [52]. This adds to the country’s burden, as the environmental damage from DSM activities could far exceed any economic benefits. Besides, the “lack of special provision” in DSM highlights the absence of differentiated responsibilities under the ISA framework. Both developing and developed countries sponsoring mining activities are held equally accountable for environmental damage caused to the ocean floor. For developing countries this creates significant challenges, as they lack the financial and technical resources of developed nations to manage and mitigate such damages [53].

The uniform application of rules creates disproportionate challenges for less-resourced states, highlighting the need for equitable provisions that consider the varying capacities of sponsoring countries. “Multiple shareholders” in DSM refers to the complexity and challenges arising from the involvement of various entities, including foreign companies and international investors, in the ownership and control of a mining operation [50]. Although a company may seem to be domestically owned, its management and financial control are often in the hands of foreign entities or shareholders. This complicates the sponsoring state’s ability to hold foreign stakeholders accountable for legal, financial, or environmental issues, increasing the risks linked to multiple levels of oversight and jurisdiction. The “knowledge gap on EAF (Economic Assistance Fund)” refers to the uncertainty surrounding the distribution of shares from DSM as compensation to terrestrial mining countries. Limited research and unresolved mechanisms for payment calculations leave the extent of compensation unclear, emphasizing the need for further study and transparent frameworks [54]. Furthermore, “loss and profitability risks” in DSM encompass several challenges, including: risk of loss and liability, where sponsoring states bear responsibility for environmental damage and must adhere to stringent international standards, potentially burdening taxpayers loss of investment [50,53], as seen in cases like PNG’s $120 million stake in Nautilus Minerals, which faced bankruptcy [55]; risk of reduced benefit, where financial and social promises of DSM often fail to materialize due to project failures and public rejection [48,56,57]; and lower success rate/profitability, as DSM lacks cost advantages over land-based mining and poses greater environmental risks, casting doubt on its long-term viability [58,59]. In DSM, the indicator “private profit over public good” reflects concerns over company monopoly and business-sided profits.

Companies with market capitalizations far exceeding the GDP of many small nations dominate the sector, leveraging global resources for legal and negotiation advantages, fostering monopolies despite the seafloor being the “common heritage of mankind” [60,61]. Moreover, cost-benefit analyses, like those conducted by MIT, reveal that miners earn nearly three times the profits compared to the public benefits distributed by the ISA [62]. Critics argue that this dynamic enriches a small elite while failing to deliver prosperity to indigenous communities or society at large [63]. Similarly, the “unacceptable royalty payment regimes” highlights concerns over inequitable compensation structures. For example, the African Group opposes time-varying royalty rates (e.g., 2%/6%) and price-varying royalties (e.g., 2%/5% to 9%), arguing that these regimes fail to provide fair compensation to humanity, do not maximize ISA revenues, and effectively subsidize DSM at the expense of land-based mining [64]. These payment structures are criticized for prioritizing industry profits over equitable benefit-sharing and global interests. Collectively, the challenges reflect the intricate economic and governance issues that may arise from engaging in DSM, where various stakeholders could encounter conflicts, regulatory ambiguities, and financial disagreements. Our results demonstrated moderate risks associated with ecological and technological costs, greenwashing arguments, political implications, comparisons between terrestrial and DSM, and tourism indicators. The findings suggest that, while these sectors are less immediately affected, they still raise significant problems that require attention.

In contrast, the scenario analysis identifies minor risks associated with a number of cross-border variables, including “social stigma,” “livelihood and employment loss,” “commercial fisheries,” “biodiversity risks,” “pollution,” “food security and safety,” and “marine habitat.” The smaller risks calculated for these indicators shows that despite the substantial challenges posed by engaging in DSM, certain aspects of environmental and social stability may remain relatively resilient to DSM impacts as a result of the assumed significant contribution of CS.

### DSM and ecosystem services

**Fig 4:**
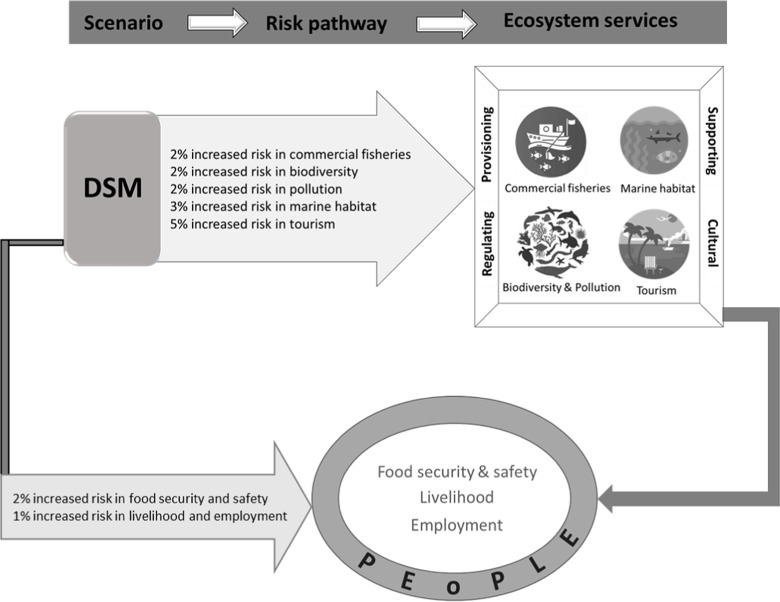
Impact of deep-sea mining (DSM) on ecosystem services and human well-being. The figure illustrates the pathways through which DSM increases risks to various ecosystem services and, in turn, affects human well-being. DSM is shown to increase risks to commercial fisheries, biodiversity, pollution levels, marine habitats, and tourism. Arrows represent the flow of these risks, moving from the scenario (DSM) to ecosystem services and subsequently impacting human well-being factors such as food security, safety, livelihood, and employment. The figure emphasizes the interconnectedness of environmental and human systems, illustrating those negative impacts on ecosystem services cascade into social and economic vulnerabilities. The percentage risk increments visually highlight the magnitude of potential impacts within each category.

The benefits that human societies obtain from ecosystems, known as ecosystem services, are essential to the well-being of both human populations and the environment. These services are commonly classified into four main categories: provisioning, supporting, regulating, and cultural services [65]. Every category represents a unique aspect of the ecosystem’s functionality, which contributes to the overall well-being and long-term viability of our planet. DSM presents serious challenges to these capabilities, specifically in the fragile and usually uncharted deep-sea ecosystems. (Fig 4) depicts the effects of DSM on distinct ecosystem services, emphasizing the related risks and benefits. The figure shows that DSM elevates risk across several ecosystem services, including a 2%, 3% and 5% increases in risks to commercial fisheries, pollution and biodiversity; to marine habitats; and to tourism, respectively. DSM threatens commercial fisheries through conflicts with fishing industries, especially in regions like the CCZ, where discharge water, heavy metal plumes, and mining noise can harm marine species and disrupt tuna fisheries [6,66]. Pollution from DSM can travel vast distances, with discharge plumes potentially reaching areas like Hawaiian and Kiribati waters, causing widespread environmental degradation [67]. Biodiversity is at risk as DSM activities may cause permanent damage to pristine habitats, leading to species loss and ecosystem fragmentation, particularly in biodiversity hotspots and deep-sea ecosystems [68,69]. Mining also disrupts marine habitats, with techniques like sediment removal and noise pollution threatening species such as the gastropod *Dracogyra subfusca* [70]. Lastly, tourism in affected regions may suffer due to the loss of unique marine species and habitats, diminishing the aesthetic and recreational value of these areas and leading to reduced tourist interest and revenue [71]. Overall, the figure suggests that engaging in DSM would likely pose significant risks to ecosystem services and human well-being.

## Discussion

The findings of this study demonstrate that DSM exacerbates risks for coastal states, a concern echoed in previous research by Jaeckel et al. [48] and Tilot et al. [56], which highlights the potential impacts on coastal communities, including disruptions to tourism due to environmental degradation and the loss or deterioration of essential ecosystem services, both on land and in marine environments. Furthermore, social indicators showing significant increases in risks, such as “violation of law,” “participatory rights,” “lack of effective control,” and “degraded reputation,” raise critical concerns about the governance of DSM. Likewise, the economic risks highlighted in this study point to potential breaches of agreements, issues with fair compensation, complex stakeholder interests, and the prioritization of private profits over public benefits. These issues highlight gaps in legal frameworks, inadequate stakeholder engagement, weak regulatory oversight, and potential damage to a country’s reputation, all of which pose serious challenges to effective DSM governance. For example, TMC has been negotiating with Tonga, Nauru, and Kiribati to allow it to explore and potentially exploit significant marine mineral resources from the Pacific Ocean [72]. In Nauru, as part of a Social Impact Assessment (SIA) study, the TMC subsidiary NORI-D conducted a broad consultation program to engage stakeholders in the development of a framework to measure and monitor its social performance [73]. This strategy represents an effort to align the company’s operations with the social and environmental expectations of the local population. On the other hand, the Civil Society Forum of Tonga (CSFT) raised concerns about Tonga’s involvement with TOML, a subsidiary of TMC [74]. The CSFT performed a study of 385 Tongans across all major island groups. The respondents regarded the ocean as a primary means of sustenance and a significant source of nourishment, which would be impacted by DSM. Yet, most of the Tongans surveyed were not informed about their government’s authorization of DSM exploration in their waters. The complexity of DSM governance stems from varying stakeholder connections to and dependencies on ocean space [75]. Therefore, to enhance transparency and accountability, the ISA needs to prioritize publishing contracts with mining proponents, opening Legal and Technical Commission meetings to the public, ensuring timely and transparent communication with a broad stakeholder community beyond its member states, and establishing clear obligations to respond to public comments and complaints [76].

Our assessment of the impacts of DSM on ecosystem services, considering economic, environmental, and social factors across various scenarios influenced by metal demand, offers a clear framework for understanding the complex effects of DSM on ecosystem service provision. For instance, DSM are associated with greater risks to vital ecosystem services, which subsequently have a negative impact on human well-being, especially in terms of food security, livelihood, and employment. DSM related impacts, including those on nutrient enrichment [77], alteration of water properties [78], oxygen depletion [79], sediment toxicity [78], removal of fauna and flora [80–82], changes in temperature and noise [83] have clear connections with the delivery of key ocean ecosystem services through their influence on ecosystem structure and processes. Furthermore, Biodiversity loss, habitat disruption, and pollution already pose significant threats to marine ecosystems [84], and their effects could be greatly intensified by DSM, which has the potential to impact the supporting services by disturbing deep-sea ecosystems that function as critical breeding and feeding grounds for numerous marine species [85].

Similarly, DSM may pose a significant impact on the cultural services derived from the ocean. In particular, DSM is likely to impact the tourism sector more severely as the degradation of deep-sea ecosystems directly, and less deep ones indirectly, may reduce their appeal and accessibility, restricting tourism opportunities and lowering economic benefits [86]. The aesthetic and cultural value of deep-sea ecosystems contribute to human well-being and should be considered when assessing DSM risks. As a whole, these impacts not only affect marine ecosystems but also the economic activities and communities that depend on them. Our findings are consistent with studies that point to the high costs and regulatory challenges inherent in DSM operations [87]. For example, if mining companies were required to cover the costs of restoration of the damage they would cause, it would significantly affect the profitability of their operations, likely turning the profits they expect to losses. Based on projections and estimates from TMC’s SEC filing, a study by Planet Tracker [87] calculated that by 2030, a company extracting polymetallic nodules over an area of 1,000 km² of the abyssal plain could potentially generate revenues of USD 4.37 billion by 2030, with operational expenses estimated to be at least USD 2.72 billion by 2030, generating a 38% profit, not accounting for additional expenses such as stock options and restoration costs. However, when the costs for ecological restoration are added as costs to the mining companies, their 38% profit becomes a large loss equivalent to almost 83% of the company’s revenues. Note that this estimated loss would even be higher if the costs of restoration and monitoring are considered [87].

Different DSM scenarios could impact the benefit-sharing mechanisms of DSM, developed within the framework of ISA [88]. The ISA is responsible for regulating DSM activities in international waters and ensuring that the benefits derived from these activities are equitably shared among all states, particularly developing countries [88]. The profits to DSM mining companies are expected to increase with the full phase operation of DSM. However, this potential increase is coupled with increased environmental, social, and economic risks, as indicated by the big increases in values of indicators such as “vulnerability to coastal states,” “degraded reputation,” “violation of law” and “loss and profitability risks” under this scenario. These elevated risks could lead to increased operational costs, legal disputes, and potential disruptions, which may ultimately diminish the overall profitability of DSM ventures. The subsequent reduction in profits would directly impact the funds available for distribution through ISA’s benefit-sharing mechanism, which is designed to distribute financial benefits to various stakeholders, including developing countries. The reduction in funds would also affect the compensation funds managed by the ISA Secretariat, which are intended to support environmental protection and compensate for any damages caused by DSM. Conversely, implementing the “Without” DSM scenario would remove these risks with likely insignificant little effect on the supply-demand equation due to the increasing deployment of circular solutions for sourcing metals.

The notable increase in environmental risks under the “With” DSM scenario observed in the study highlights the critical need for comprehensive environmental impact assessments, the development of clear and enforceable environmental management guidelines, and the exploration of alternative sources of high-demand metal supplies, such as the “circular solutions”. Up to 90% of the world’s electronic waste is illegally traded or dumped [89]. This highlights the significant loss of valuable metals, emphasizing the immense potential for policies aimed at enhancing global resource efficiency and stressing the need to prioritize solutions such as urban mining over DSM. Policies should incentivize industries to adopt circular practices by providing financial support for research into better recycling technologies and promoting regulatory frameworks that encourage resource recovery [90]. Moreover, strategies that support the efficient use of resources and the development of closed-loop systems can contribute to reducing reliance on virgin materials from both terrestrial and marine sources. Through these circular solutions, societies can continue to meet their technological and industrial needs without compromising environmental integrity and future generations’ access to natural resources.

## Conclusion

This study demonstrates that environmental factors hold the highest centrality in deep-sea mining (DSM) dynamics, revealing their dominant influence relative to social and economic components. The substantial environmental risks associated with DSM—including biodiversity loss, pollution, and irreversible habitat disturbance—corroborate the findings of previous studies, which have consistently emphasized the fragility of deep-sea ecosystems and their slow recovery rates [91,92]. Our results further align with research indicating that DSM not only poses direct ecological threats but also introduces significant socio-economic uncertainties, particularly for coastal states and communities dependent on marine ecosystem services [93].

While proponents of DSM argue that it offers potential economic benefits, our findings suggest that these advantages can be easily negated by the compounded risks to ecosystem services, governance structures, and economic stability. The elevated risks under the “With DSM” scenario—such as increased vulnerability of coastal economies, disruptions to social governance, and challenges to equitable resource distribution—reinforce concerns raised in previous literature about the uncertain financial viability of DSM once environmental restoration costs and social externalities are accounted for [94]. The potential for declining profits, coupled with the complexities of implementing the ISA’s benefit-sharing mechanisms, further questions the economic justification for DSM.

Given these risks, a fundamental question arises: Do we truly need deep-sea minerals to meet future resource demands? Our findings suggest that DSM is not an inevitable necessity. Instead, alternative strategies—rooted in circular economy principles—offer viable pathways to securing critical metals while mitigating environmental and socio-economic harm. Expanding metal recovery through enhanced recycling, reusing electronic waste, and advancing urban mining technologies can significantly reduce reliance on virgin mineral extraction [95]. Recent studies have demonstrated that with improved policies, such as extended producer responsibility [96] and investments in closed-loop production systems, the supply gap for key metals can be narrowed without resorting to destructive seabed mining [97].

From a policy perspective, regulators, including the ISA, must take a proactive role in reassessing DSM’s long-term sustainability and prioritizing alternative resource strategies. A key recommendation is the integration of circular economy principles into global mineral governance frameworks, encouraging responsible sourcing and technological innovations that diminish dependency on newly mined materials. By fostering investment in sustainable supply chains and promoting the reuse of existing mineral stocks, policymakers can better align resource security with environmental stewardship. Ultimately, the shift toward a more resource-efficient economy can not only meet humanity’s mineral demands but also safeguard oceanic ecosystems, ensuring that future generations inherit a habitable and biodiverse planet.

## Supporting information

S1 File **S1 Table.** List of environmental indicators. **S2 Table.** List of economic indicators. S3 Table. List of social indicators. S**4** Table. Score calculation for circular solutions. **S4 Diagram.** Interconnected risk assessment indicators of deep-sea mining.
(DOCX)
