# Supplementary material for: Deep-sea mining and its risks for social-ecological systems: Insights from simulation-based analyses
Source: PLoS One. 2025 Mar 28;20(3):e0320888. doi: 10.1371/journal.pone.0320888 (PMC11952235; doi:10.1371/journal.pone.0320888)
Supplement: S1 File — S1 Table. List of environmental indicators. S2 Table. List of economic indicators. S3 Table. List of social indicators. S4 Table. Score calculation for circular solutions. S4 Diagram. Interconnected risk assessment indicators of deep-sea mining. (DOCX) [file pone.0320888.s001.docx]

**S1 Table: List of environmental indicators**

| **Risk Indicators** | **Components** | **Description** |
| --- | --- | --- |
| Biodiversity risk | Loss of Species and ecosystem | DSM is predicted to result in intense damage to few of the world’s pristine habitats, many of which are ecologically and biologically significant areas with biodiversity hotspots [1]. The most direct DSM impact would be species loss/fragmentation, loss of ecosystem structure and function. Furthermore, the damages caused to deep-sea ecosystems are most likely to be permanent [2]. |
|  | Biodiversity threats | Mining affects the cetaceans and tourism industry which is highly dependent on whale watching. Mining on environment like this with such high degree of uniqueness and endemism will impact the biodiversity greatly, which will also be difficult to reverse [3]. |
|  | Marine ecosystems and biodiversity risk | While the Norwegian government insists that it can conduct deep-sea mining in a sustainable way, critics say that these activities will put the marine ecosystems and biodiversity at risk [4]. |
| Vulnerability to coastal states | Vulnerability to coastal states | Mining is about to threaten the small island developing states (SIDS) surrounding the CCZ, which have already been threatened by continuous rise in sea levels [5]. As sea levels rise, additional stress from mining activities could lead to increased coastal erosion and habitat loss, exacerbating the challenges these nations face [6]. This intersection of deep-sea mining sector and climate change thereby tend to complicate the scenario for SIDS. |
| Pollution | Pollution outreach from area to adjacent states | Pollution discharged by TMC in its Tonga sponsored license area is expected to reach Hawaiian and Kiribati waters in just three months. The mining operations are expected to destroy an area of seabed equivalent to the land area of the whole of Hawaii in the 30-year license period [7], if granted, given the potent of discharge plume to travel over 1,400km. DSCC [5] further highlights that the word adjacent, may not cover all the affected states and therefore potentially affected states may therefore not be adjacent, indicating much broader implications of DSM on the coastal states. |
| Troublesome sea life | Disturbing sea life of neighboring territories | The "troublesome sea life" indicator serves to highlight the potential disturbances to marine ecosystems that may arise from DSM operations. These disruptions can appear as sediment plumes, poisonous metals, and 'dust clouds,' all of which can harm marine creatures [8–10]. |
| Knowledge gaps | Knowledge gaps and inadequate research | The Norway Institute of Marine Research (IMR) said that the government had made assumptions from a small area of research and applied it to the whole area planned for drilling. There is currently not enough knowledge needed to extract minerals from the seabed in the manner required and a period of 5 to 10 years of research into impacts on species will be needed, to know the exact impacts of DSM. These knowledge gaps on this nascent sector might demand more investments from the private sectors to carry out research on the deep marine environments [11]. |
|  | Poor scientific knowledge and unforeseen impacts on marine ecosystem | Opponents of DSM emphasize that scientific knowledge of the ecosystem in deep sea and other marine environments are poorly understood. Moreover, DSM relies on naïve/untested technologies that may result in unforeseen and long-lasting impacts to the marine ecosystems by changing the characteristics of the seabed and leading to habitat changes [12]. |
| Greenwashing argument | Greenwashing argument | The urgency of mineral scarcity necessitates a shift towards recycling as a primary source of critical minerals, rather than relying on new extraction methods that could exacerbate environmental degradation [13]. By prioritizing DSM which tends to be extractive and unsustainable, there is a risk that policymakers and industries may overlook the potential of recycling existing materials, which could provide a more sustainable and less environmentally damaging solution to mineral shortages [8]. |
| Ambiguous guidelines | Lack of clear guidelines in practical application of precautionary principle | Papua New Guinea (PNG) still lacks a clear set of guidelines in implementing the precautionary principle within their regulatory framework. It was evidenced through the solwara 1 project, where they didn't apply the precautionary principle in practical aspects though uncertainty on environmental effects existed along with lack of data [14]. |
| Habitat risk | Ocean habitat risk | Mining techniques may cause significant noise and light pollution, disrupting the habitats of organisms’ dependent on seabed nodules [2] as they involve the removal of large volumes of sediment and rock. This further threatens the survival of certain biota, eg: the gastropod *Dracogyra subfusca*, which is already facing risks due to ongoing exploration activities in hydrothermal vent field [15]. |

**S2 Table: List of economic indicators**

| **Risk Indicators** | **Components** | **Description** |
| --- | --- | --- |
| Private profit over public good | Company monopoly | Mining companies have market capitalization values, greatly in excess of many small countries annual GDP and can draw upon global sources for legal and negotiation purposes [16]. Though the seafloor tends to be the common heritage of mankind, the current scenarios reflect the formation of monopoly [17]. |
|  | Business sided profits | The estimates of the cost benefit analysis models (MIT) indicate a higher benefit to miners (nearly thrice) than that of the ISA from where the benefits flow to mankind as a whole. The opponents and environmental warriors claim that DSM would benefit only a small number of people, making the rich richer and in no way will bring prosperity to the indigenous communities [18,19]. |
| Risk on commercial fisheries | Impact on fisheries | DSM in the Pacific could have irreversible impacts on ocean-dependent communities, particularly the world's largest tuna industry. Contamination of seafood near mining sites and increased activities near spawning areas could affect fisheries stocks. Due to the high migratory nature of tuna, the impacts in one region have the potential to be transmitted to other regions that are unaffected by mining but rely on the fishing business [20]. |
|  | Threats to commercial fisheries | Deep-sea mining impacts may intersect with other industries, such as the fishing industry which is significant for the economy of many countries. Conflict between fisheries and DSM will occur given the existing spatial overlap within and around CCZ. The suspended sediments and discharge waters with heavy metals impacting pelagic communities will have direct consequences on the fishing industries if the target species are directly or indirectly impacted. DSM impacts tuna fisheries through discharge water and plumes containing heavy metals, mining noise and increased density of mining vessels operation which will limit fishing vessels operation [21]. |
| Ecological cost | Cost for ecological impacts | The estimated cost associated with unavoidable ecological impacts to benthic communities due to mining activities ($500,000) and unplanned releases are very high ranging up to $28.6 million [22]. The question of who is to bear this burden in case of its occurrence remains unanswered till now. |
|  | Higher costs associated with environmental degradation | DSM is said to result in increased environmental and post closure costs, with the profitability expected to be marginal. Moreover, the costs linked to offsetting environmental degradation in the deep- seabed including post-closure remain largely unknown, highlighting the risks on investors to be very high [23]. |
| Technological cost | Technological advancements | Innovative technologies such as sodium-ion batteries can potentially mitigate demand for critical minerals, together with the rise of mature battery chemistries requiring lower amounts of critical metals, such as lithium iron phosphate (LFP). While low critical mineral prices help bring battery costs down, they also imply lower cash flows and narrower margins for mining companies [24]. |
|  | Constraints in technology development and financial potential | Developing states, including Least Developed Countries (LDCs), Landlocked Developing Countries (LLDCs), and Small Island Developing States (SIDS), face significant hurdles due to a lack of advanced technology and financial resources, which, combined with revenue risks and acute poverty, discourage investment in DSM [25,26]. |
| Loss and profitability risks | Risk of loss and liability | The risks of losses and liabilities in a developing country's business scheme governed by international laws, regulations, and competencies are of the same standards as developed countries. For example, under the current rules, a developing country like Tonga is liable for the damage if it fails to fulfil its obligations as a sponsor, for which it has to maintain highest standards of environmental safety. This would ultimately put the Tongan taxpayers at burden [27,28]. |
|  | Loss of investment | Officials in PNG emphasize that the country had a 15% stake in Nautilus Minerals prior to its bankruptcy. PNG still stands to lose all of that reported $120 million investment in the company, unless it terminates the license [29]. |
|  | Risk of reduced benefit | Allowing for DSM in the sulfide deposits in PNG has the potential to improve the well-being of the people in these nations [22]. However, Nautilus Minerals’ failed DSM project in PNG has encountered financial challenges and public rejection as well [30–32]. |
|  | Lower success rate/profitability | The success rate of DSM is doubtful as it does not tend to have significant cost advantage over land-based mining at current and projected factor prices [33]. It is also critiqued to lead to worse impacts than land-based mining for its operation in new environments [34]. |
| Tourism | Impact on tourism industry | Mentioned as one among the potential impacts of DSM on people, the environmental degradation resulting from the mining activities might have subsequent impacts on the tourism industry [31]. The potential loss of unique marine species and habitats can diminish the aesthetic and recreational value of these areas, leading to decreased tourist interest and revenue [35]. |
| Royalty payment regimes | Unacceptable royalty payment regimes | The African Group does not support the time varying royalty including rates of 2%/6% or the price varying royalty if the suggested rates are proposed to be 2%/5% to 9% in the royalty payment regimes. It is because, these payment regimes neither results in fair compensation to mankind nor maximise the ISA revenues and subsidise DSM relative to land-based mining [36]. |
| Knowledge gap on EAF | The Economic Assistance Fund: Lacking knowledge | The percentage of shares from DSM that will be available for distribution as a compensation to the terrestrial mining countries from the EAF (Economic assistance fund) is still under negotiation/discussion, given the lack of research to date on this matter and the conceptual and practical difficulties associated with payment calculation. It is therefore still unknown to what extent they will be compensated for the economical loss [37]. |
| Terrestrial mining vs DSM | Competing interest with terrestrial mining countries | The countries producing DSM minerals will be competing with the major African states that are land-based producers of copper and cobalt, including Zambia, Botswana, Morocco, Madagascar, Gabon, South Africa, Namibia, Democratic republic of Congo and Zimbabwe. Surplus mineral resources in the market and fall down of prices will adversely affect the economy of these land-based mining countries [38,39]. |
| Multiple shareholders | Complexity with multiple shareholders | Though on paper, TOML is Tongan, it is financially controlled by Canada and management wise by both Canadians and Australians [27]. |
| Lack of special provision | Lack of specific clause | Whether it is developing or developed countries, the rules apply equally for the sponsoring countries and there is no special provision under international laws in ISA for the developing country like Tonga, where they are completely responsible for mining damage caused to ocean floor on the same level of culpability as developed countries [28]. |
| Contractual violation | Risk economic loss due to contractual violation | In case if the subsidiary, Tonga Offshore Mineral Limited, violated its contractual obligation with the ISA, Tonga might be left with a heavy bill to pay which is an additional concern for the country as the environmental damage itself far outweighs the benefits [40]. |

**S3 Table: List of social indicators**

| **Risk Indicators** | **Components** | **Description** |
| --- | --- | --- |
| Political implications | Political volatility | The "political implications" risk indicator examines how DSM activities might influence geopolitical and security dynamics. For example, the Clarion-Clipperton Zone (CCZ), located in international waters, is strategically significant for countries such as Russia, China, and the US. Political volatility between these countries could lead to geopolitical tensions [41]. |
|  | Geopolitical implications | Norway’s decision of entering DSM could have geopolitical implications. The launch of Arctic mining would increase the international competition for resources and change the dynamics of the region [8]. |
| Social stigma | Loss of cultural and heritage values | Deep sea mining threatens the cultural, heritage and spiritual values associated with pristine ocean and sentimental beliefs of the indigenous communities. These communities believe that the deep sea and its seabed are intimately connected to humanity despite the geographical distances involved. According to them, DSM disturbs the sense of who they are including the spirits that inhabit their culture and beliefs [42]. |
|  | Lack of societal acceptance | Different aspects of social acceptability have contributed to conflict situations and ultimately the demise of the DSM project, as happened in the case of Solwara 1. Financial issues related to foreign companies, asymmetry in power balance between pacific states and global business, governance challenges, lack of community and stakeholder acceptance were the critical factors in the negative perception of societal acceptance [43]. |
|  | Lack of consent from citizen | There are no governance procedures in Tonga where the state conscientiously informs the public about the project happenings to gain their consent, particularly those which might affect their land and territories. The government policy also fails to ask public opinion on denationalizing deep sea minerals when calculating the cost and benefits to the country [28] . |
| Food security and safety |  | Although the fishing activities of most countries have minimal overlap with the proposed mining sites, there are a number of countries, particularly developing or small island states, that could experience significant impacts on their fishing yields. Research reveals that in certain regions, up to 10 percent of the catch may be affected, leading to substantial decreases in the economy and food security of these countries [44]. Seafood caught near mining sites could be contaminated with toxic metals impacting seafood safety [45,46]. |
| Lack of effective control |  | Sponsoring states must have effective control over the companies they sponsor, but the ISA has not explicitly defined what that means. For eg: TOML’s exploration contract says that if control changes, it must find a new sponsoring state [27]. |
| Violation of the law |  | a. Norway’s decision on deep sea mining could be a “violation of the law” due to a lack of scientific evidence needed to assess the environmental impacts of future mining activities, which is legally needed for such decisions to be made [4].  b. Norwegian law firm Wilkborg Rein said that passing the bill with an inadequate environmental assessment could violate not only the country’s own laws on environmental protection, but also European and international laws [8]. |
| A degraded reputation |  | Norway has become the first country in the world to open up its seabed for commercial DSM, despite warning from scientists and experts on environmental concerns. This decision of DSM thereby might act as an irrevocable black mark on Norway’s reputation as a responsible ocean state [47]. |
| Livelihood and employment loss | Livelihood and employment loss | Marine mining, in the short to mid-term is relatively a small part of the total blue economy [23]. As DSM activities expand, they pose risks to marine ecosystems challenging the sustainability of other blue economic sectors, particularly fisheries and tourism, which often relies on healthy coastal and marine ecosystem. This in turn leads to significant implications for the livelihood and employment opportunities of communities dependent on these sectors due to the economic hardships they face upon mining, resulting in increased unemployment and social instability [6]. |
| Participatory rights | Inadequacy of Participatory rights | The "participatory rights" risk indicator points out the inadequacy in ensuring procedural rights under the current legal regime for DSM. Integrating indigenous people into DSM management is not just a mere issue of a ‘participatory approach’ as acknowledged in various international projects and established in some regulations of Pacific States; but is the very foundation of holistic custom-based relationships. Yet, in state like PNG, there is a notable lack in participation of stakeholders, common public and fishing communities during the decision-making process for DSM projects, which would be philosophically problematic for Oceanian society [31,48]. |

**S4 Diagram. Interconnected risk assessment indicators of deep-sea mining**


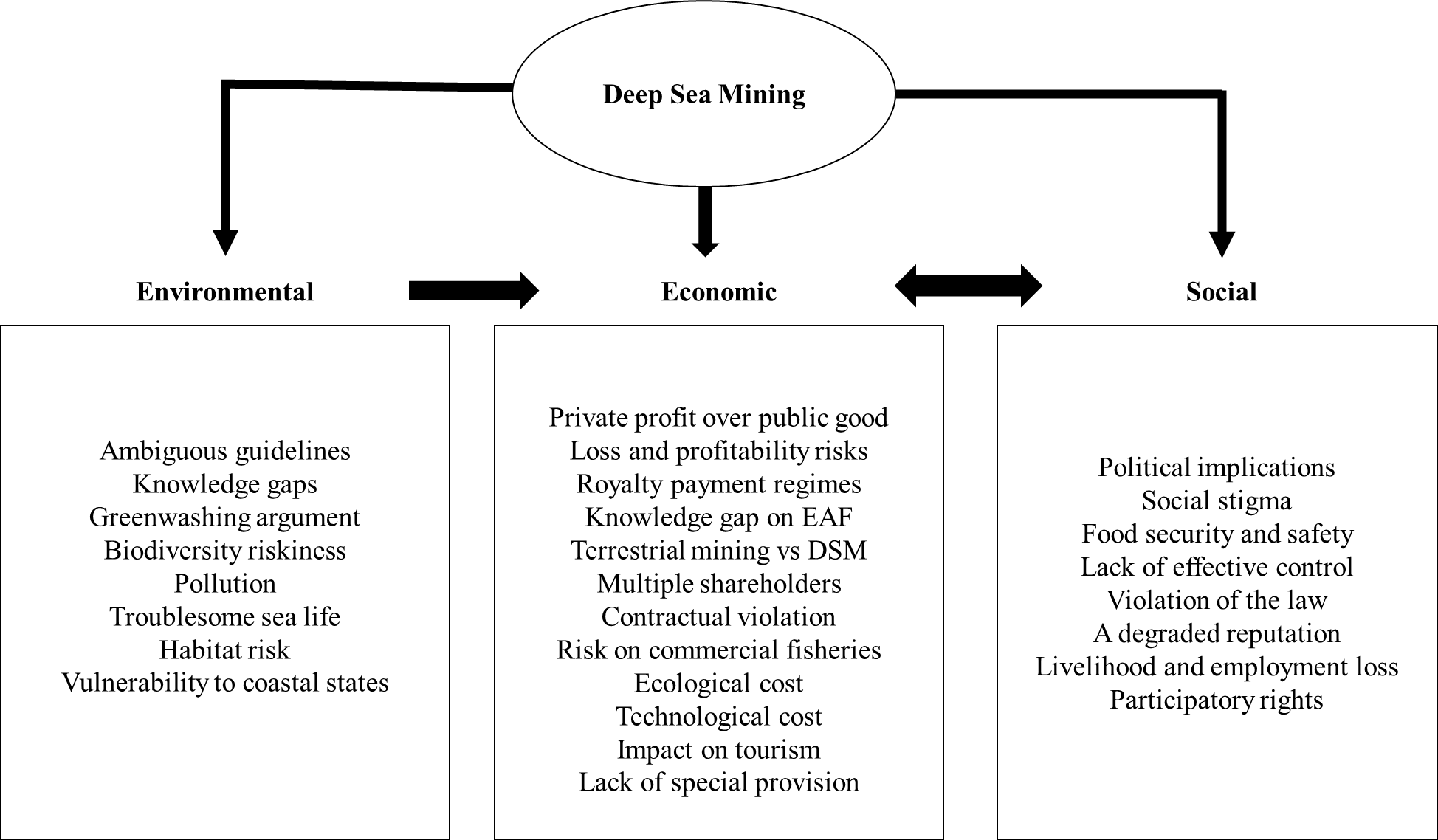


**S4 Table: Score calculation for circular solutions**

| **No** | **Ref** | **FS** | **TM** | **MN** | **BD** | **CTS** | **PLN** | **SL** | **HB** | **PL** | **SS** | **FSS** | **VL** | **LE** |
| --- | --- | --- | --- | --- | --- | --- | --- | --- | --- | --- | --- | --- | --- | --- |
| 1 | [49] | 1 |  |  |  |  | 1 |  |  |  |  | 1 |  | 1 |
| 2 | [50] | 1 | 1 |  | 1 |  | 1 | 1 | 1 |  |  | 1 |  | 1 |
| 3 | [51] |  |  |  |  |  |  |  |  |  |  |  |  | 1 |
| 4 | [52] | 1 |  |  | 1 |  | 1 |  | 1 |  | 1 | 1 |  | 1 |
| 5 | [53] | 1 |  |  | 1 |  | 1 | 1 | 1 | 1 | 1 | 1 |  | 1 |
| 6 | [54] | 1 | 1 |  | 1 | 1 | 1 | 1 | 1 | 1 | 1 | 1 | 1 | 1 |
| 7 | [55] | 1 | 1 |  |  | 1 | 1 | 1 | 1 | 1 | 1 | 1 |  | 1 |
| 8 | [56] |  |  | 1 |  |  | 1 |  |  |  |  |  |  |  |
| 9 | [57] | 1 |  | 1 | 1 | 1 | 1 | 1 |  |  | 1 |  |  | 1 |
| 10 | [58] | 1 |  | 1 | 1 |  | 1 |  |  | 1 | 1 | 1 | 1 | 1 |
| 11 | [59] |  |  |  |  |  | 1 |  |  |  |  |  |  | 1 |
| 12 | [60] | 1 | 1 |  | 1 | 1 | 1 | 1 | 1 |  | 1 | 1 |  |  |
| 13 | [61] | 1 |  |  | 1 | 1 | 1 | 1 | 1 | 1 | 1 | 1 |  | 1 |
| 14 | [62] |  |  |  | 1 |  |  |  |  |  | 1 | 1 |  | 1 |
| 15 | [63] | 1 | 1 | 1 | 1 | 1 | 1 | 1 | 1 | 1 | 1 | 1 |  | 1 |
| 16 | [64] | 1 | 1 | 1 | 1 | 1 | 1 | 1 | 1 | 1 | 1 | 1 | 1 | 1 |
| 17 | [65] | 1 | 1 |  | 1 |  | 1 |  |  | 1 | 1 | 1 | 1 | 1 |
| 18 | [66] | 1 | 1 | 1 | 1 |  | 1 | 1 | 1 | 1 | 1 | 1 | 1 | 1 |
| 19 | [67] | 1 | 1 |  | 1 | 1 | 1 | 1 | 1 |  | 1 | 1 | 1 | 1 |
| 20 | [68] | 1 |  | 1 | 1 |  | 1 | 1 | 1 | 1 | 1 | 1 |  | 1 |
| 21 | [49] | 1 |  | 1 |  |  |  |  |  |  |  | 1 |  | 1 |
|  | Total | 17 | 9 | 8 | 15 | 8 | 18 | 12 | 12 | 10 | 15 | 17 | 6 | 19 |
|  | Score | 0.81 | 0.43 | 0.38 | 0.71 | 0.38 | 0.86 | 0.57 | 0.57 | 0.48 | 0.71 | 0.81 | 0.29 | 0.90 |

Ref: References, FS: fisheries, TM: tourism, MN: mining, BD: biodiversity, CTS: coastal states, PLN: pollution, SL: sea life, HB: habitat, PL: political, SS: social stigma, FSS: food security and safety, VL: violation of the law, LE: livelihood and employment.

**References**

1. Amon DJ, Levin LA, Metaxas A, Mudd GM, Smith CR. Heading to the deep end without knowing how to swim: Do we need deep-seabed mining? One Earth. 2022;5: 220–223. doi:10.1016/j.oneear.2022.02.013

2. IUCN. The impact of deep sea mining on biodiversity, climate and human cultures. 2024. Available: https://www.iucn.nl/en/news/the-impact-of-deep-sea-mining-on-biodiversity-climate-and-human-cultures/

3. Sheikh S El. Tonga seeks support to determine the best way forward to explore seabed minerals while protecting its marine environment. 2018. Available: https://www.google.com/search?q=Tonga+seeks+support+to+determine+the+best%0D%0Away+forward+to+explore+seabed+minerals%0D%0Awhile+protecting+its+marine+environment&sca_esv=da56b67854fd2398&rlz=1C1RXMK_enIN977IN977&ei=w4DyZba4IuiUseMPx

4. Alberts EC. Really a sad day’ as Norway votes to allow deep-sea mining in Arctic waters. 2024. Available: https://news.mongabay.com/2024/01/really-a-sad-day-as-norway-votes-to-allow-deep-sea-mining-in-arctic-waters/#:~:text=%25E2%2580%259CIt’s%2520really%2520a%25

5. DSCC. Tonga – Key Statements. 2022. Available: Tonga Archives - The DSCC ISA Tracker (deep-sea-conservation.org)

6. Ramirez-Llodra E, Tyler PA, Baker MC, Bergstad OA, Clark MR, Escobar E, et al. Man and the Last Great Wilderness: Human Impact on the Deep Sea. PLoS One. 2011;6: e22588. doi:10.1371/JOURNAL.PONE.0022588

7. Blue Peril. New Modelling predicts Deep Sea mining by Tonga and The Metals Company to pollute Hawaii and Kiribati Waters. 2022. Available: https://dsm-campaign.org/blue-peril-media-advisory/

8. Gkritsi E. Norway’s Deep-Sea Mining Decision Is a Warning. 2024. Available: https://www.wired.com/story/norway-deep-sea-mining-arctic-svalbard-batteries-environment/

9. Putol R. Deep-sea mining forms “dust clouds” that devastate marine life. 2024. Available: https://www.earth.com/news/deep-sea-mining-fo

10. Washburn TW, Simon-Lledó E, Soong GY, Suzuki A. Seamount mining test provides evidence of ecological impacts beyond deposition. Current Biology. 2023;33: 3065-3071.e3. doi:10.1016/j.cub.2023.06.032

11. Stallard E. Deep-sea mining: Norway approves controversial practice. 2024. Available: https://www.bbc.com/news/science-environmen

12. Benkenstein A. Seabed Mining: Lessons from the Namibian Experience. 2014.

13. Giurco D, Littleboy A, Boyle T, Fyfe J, White S. Circular Economy: Questions for Responsible Minerals, Additive Manufacturing and Recycling of Metals. Resources . 2014;3: 432–453. doi:10.3390/RESOURCES3020432

14. Olorundami F. Developing an international legal framework for the environmental regulation of deep sea mining: a comparative analysis of the deep sea mining regulations of Papua New Guinea, the Cook Islands and the United-States. Economic challenge and new maritime ris. 2017.

15. Thomas EA, Böhm M, Pollock C, Chen C, Seddon M, Sigwart JD. Assessing the extinction risk of insular, understudied marine species. Conservation Biology. 2022;36: e13854. doi:10.1111/COBI.13854

16. Petterson MG, Tawake A. The Cook Islands (South Pacific) experience in governance of seabed manganese nodule mining. Ocean Coast Manag. 2019;167: 271–287. doi:10.1016/J.OCECOAMAN.2018.09.010

17. Groch S. It’s bloody El Dorado’: Undersea riches to be reaped by the few. 2023. Available: https://www.smh.com.au/world/oceania/it-s-bloody-el-dorado-undersea-riches-to-be-reaped-by-the-few-20230713-p5do2v.html

18. DSCC. Deep sea mining: What do we stand to lose. 2020. Available: https://deep-sea-conservation.org/wp-content/uploads/2024/01/DSCC_FactSheet6_DSM_LOSE_4pp_OCT_17_23.pdf

19. Mesulam J, Baxter C, Lowrey N. Sinking Seabed Mining: Papua New Guinean, Australian and New Zealand civil society welcome ban on seabed mining in Northern Territory. 2021. Available: https://miningwatch.ca/news/2021/2/11/sinking-seabed-mining-papua-new-guinean-australian-and-new-zealand-civil-society

20. Grient J Van der. Seabed mining could sink the fishing industry. 2023. Available: https://www.downtoearth.org.in/mining/seabed-mining-could-sink-the-fishing-industry-90430

21. Amon DJ, Palacios-Abrantes J, Drazen JC, Lily H, Nathan N, van der Grient JMA, et al. Climate change to drive increasing overlap between Pacific tuna fisheries and emerging deep-sea mining industry. npj Ocean Sustainability. 2023;2: 1–8. doi:10.1038/s44183-023-00016-8

22. Wakefield JR, Myers K. Social cost benefit analysis for deep sea minerals mining. Mar Policy. 2018;95: 346–355. doi:10.1016/J.MARPOL.2016.06.018

23. Löf A, Ericsson M, Löf O. Marine mining and its potential implications for low- and middle-income countries. 2022 [cited 29 Aug 2024]. doi:10.35188/UNU-WIDER/2022/303-1

24. Global EV Outlook. Moving towards increased affordability. 2024. Available: https://www.iea.org/reports/global-ev-outlook-2024

25. Egede E. Africa and the deep seabed regime: politics and international law of the common heritage of mankind. Springer Science and Business Media B.V.; 2011.

26. Samuel olatunde Popoola. Potential risks of developing countries in the process of exploration and extraction of deep-sea mineral resources. Journal of Atmosphere and Oceanography Environment. 2023;11.

27. Morse I. What if seabed mining goes wrong? 2021. Available: https://greenrocks.substack.com/p/seabed-mining-tonga-deepgreen-evs-isa

28. Brown Pulu T. Off the deep end: Tonga’s continental shelf politics. Te Ara Poutama - the Faculty of Maori and Indigenous Development, Auckland University of Technology; 2014. Available: https://hdl.handle.net/10292/7237

29. Cannon J. Deep-sea mining project in PNG resurfaces despite community opposition. Mongabay. 2023. Available: https://news.mongabay.com/2023/08/deep-sea-mining-project-in-png-resurfaces-despite-community-opposition/

30. Tilot V, Willaert K, Guilloux B, Chen W, Mulalap CY, Gaulme F, et al. Traditional Dimensions of Seabed Resource Management in the Context of Deep Sea Mining in the Pacific: Learning From the Socio-Ecological Interconnectivity Between Island Communities and the Ocean Realm. Front Mar Sci. 2021;8: 637938. doi:10.3389/FMARS.2021.637938/BIBTEX

31. Jaeckel A, Harden-Davies H, Amon DJ, van der Grient J, Hanich Q, van Leeuwen J, et al. Deep seabed mining lacks social legitimacy. npj Ocean Sustainability. 2023;2: 1–4. doi:10.1038/s44183-023-00009-7

32. Filer C, Gabriel J. How could Nautilus Minerals get a social licence to operate the world’s first deep sea mine? Mar Policy. 2018;95: 394–400. doi:10.1016/J.MARPOL.2016.12.001

33. Donges J. The economics of deep-sea mining. Springer Science and Business Media B.V.; 2012.

34. Hallgren A, Hansson A. Conflicting Narratives of Deep Sea Mining. Sustainability. 2021;13: 5261. doi:10.3390/SU13095261

35. Thompson KF, Miller KA, Currie D, Johnston P, Santillo D. Seabed mining and approaches to governance of the deep seabed. Front Mar Sci. 2018;5: 395219. doi:10.3389/FMARS.2018.00480/BIBTEX

36. African Group. International Seabed Authority: African Group Submission on the Payment Regime for Deep- sea Mining in the Area, Executive summary. 2022. Available: https://www.isa.org.jm/wp content/uploads/2022/12/African_Group_Submission_Payment_Regime.pdf

37. Wilde D, Lily H, Craik N, Chakraborty A. Equitable sharing of deep-sea mining benefits: More questions than answers. Mar Policy. 2023;151: 105572. doi:10.1016/J.MARPOL.2023.105572

38. Cuyvers L, Berry W, Kristina G, Torsten T, Caroline W. Deep seabed mining: a rising environmental challenge. International Union for Conservation of Nature (IUCN) . 2018. doi:10.2305/IUCN.CH.2018.16.EN

39. ISA Technical Study No 32. Study of the potential impact of Polymetallic nodules production in the Area on the economies of developing land based producers of those metals which are likely to be most seriously affected. 2022. Available: https://www.isa.org.jm/wp-content/uploads/2022/12/ISA-Technical-Study-32.pdf

40. RNZ News. Tongans question government plans for seabed mining. 2020. Available: https://www.google.com/search?q=Tongans+question+government+plans+for+seabedmining&rlz=1C1RXMK_enIN977IN977&oq=Tongans+question+government+plans+for+seabedmining&gs_lcrp=EgZjaHJvbWUyBggAEEUYOdIBCTEzNjJqMGoxNagCALACAA&sourceid=chrome&ie=UTF-8

41. Sidhu A. Troubled Waters: The Geopolitics of Deep-Sea Mining. 2023. Available: https://www.geopoliticalmonitor.com/troubled-w

42. Childs J. Deep sea mining threatens indegenous culture in Papua New Guinea. The Conservation. 2019;19. Available: https://theconversation.com/deep-sea-mining-threatens-indigenous-culture-in-papua-new-guinea 112012#:~:text=Thus%2C the prospect of deep,to the sea and land.&text=out there…

43. van Putten EI, Aswani S, Boonstra WJ, De la Cruz-Modino R, Das J, Glaser M, et al. History matters: societal acceptance of deep-sea mining and incipient conflicts in Papua New Guinea. Maritime Studies. 2023;22: 1–17. doi:10.1007/S40152-023-00318-0/FIGURES/3

44. Sousa E De. Ocean mining’s energy potential could put global fisheries at risk. 2021.

45. van der Grient JMA, Drazen JC. Potential spatial intersection between high-seas fisheries and deep-sea mining in international waters. Mar Policy. 2021;129: 104564. doi:10.1016/J.MARPOL.2021.104564

46. Hauton C, Brown A, Thatje S, Mestre NC, Bebianno MJ, Martins I, et al. Identifying toxic impacts of metals potentially released during deep-sea mining-A synthesis of the challenges to quantifying risk. Front Mar Sci. 2017;4: 302120. doi:10.3389/FMARS.2017.00368/BIBTEX

47. Miranda Bryant. Norway votes for deep-sea mining despite environmental concerns | Deep-sea mining | The Guardian. 2024 [cited 29 Aug 2024]. Available: https://www.theguardian.com/environment/2024/jan/09/norway-set-to-approve-deep-sea-mining-despite-environmental-concerns

48. Hamley GJ. The implications of seabed mining in the Area for the human right to health. Rev Eur Comp Int Environ Law. 2022;31: 389–398. doi:10.1111/REEL.12471

49. Lakra WS, Krishnani KK. Circular bioeconomy for stress-resilient fisheries and aquaculture. Biomass, Biofuels, Biochemicals. 2022; 481–516. doi:10.1016/B978-0-323-89855-3.00010-8

50. Grimstad SMF, Ottosen LM, James NA. Innovative solutions to tracking waste. Marine Plastics. 2023; 274.

51. IISD & SITRA. Effects of the Circular Economy on Jobs2020. 2020. Available: https://www.iisd.org/publications/circular-economy-jobs

52. Gower R, Schröder P. Virtuous Circle: how the circular economy can create jobs and save lives in low and middle-income countries. Institute of Development Studies and Tearfund, UK. 2016.

53. Cunningham R, Barclay K, Jacobs B, Sharpe S, McClean N. Circular Economy Opportunities for the Fisheries and Aquaculture Sector in Australia. 2022.

54. World Bank, United Nations Department of Economic and Social Affairs. The potential of the blue economy: increasing long-term benefits of the sustainable use of marine resources for small island developing states and coastal least developed countries. 2017

55. Burch MV, Rigaud A, Binet T, Barthélemy C. Circular economy in fisheries and aquaculture areas. Vertigo Lab, https://webgate. ec. europa. eu/fpfis/cms/farnet2/sites/farnet/files/publication/en_farnetguide17. pdf. 2019.

56. Binnemans K, Jones PT, Blanpain B, Van Gerven T, Yang Y, Walton A, et al. Recycling of rare earths: a critical review. J Clean Prod. 2013;51: 1–22. doi:10.1016/J.JCLEPRO.2012.12.037

57. Mesut S. Blue economy and blue Ocean strategy. J Ecol Nat Resour. 2021;5.

58. MacArthur E. Towards the circular economy. J Ind Ecol. 2013;2: 23–44.

59. Reuter M, Hudson C, Van Schaik A, Heiskanen K, Meskers C, Hagelüken C. Metal recycling: Opportunities, limits, infrastructure. A report of the working group on the global metal flows to the international resource panel. 2013 Feb 18.

60. Ten Brink P, Schweitzer JP, Watkins E, Howe M. Plastics marine litter and the circular economy. A briefing by IEEP for the MAVA Foundation. 2016 Oct.

61. Ellen MacArthur Foundation. The Nature Imperative: How the circular economy tackles the biodiversity loss. In: 2021.

62. Jensen-Cormier S, Vaughan S, Gass P. Estimating employment effects of the circular economy. 2018 [cited 30 Aug 2024]. Available: https://policycommons.net/artifacts/614323/estimating-employment-effects-of-the-circular-economy/1594593/

63. Günther J, Manshoven S, Paleari S, Fuchs G, Carré A, Fischer-Bogason R, Nielsen T. Circular Economy and Biodiversity*.* European Topic Centre on Circular economy and resource use. 2023.

64. European Commission. EU Biodiversity Strategy for 2030*:* Bringing nature back into our lives (Communication from the Commission to the European Parliament, the Council, the European Economic and Social Committee, and the Committee of the Regions). Brussels: European Commission. 2020.

65. Ghisellini P, Cialani C, Ulgiati S. A review on circular economy: the expected transition to a balanced interplay of environmental and economic systems. J Clean Prod. 2016;114: 11–32. doi:10.1016/J.JCLEPRO.2015.09.007

66. Herrador M, Van ML. Circular economy strategies in the ASEAN region: A comparative study. Science of The Total Environment. 2024;908: 168280. doi:10.1016/J.SCITOTENV.2023.168280

67. Verma M. Deep Sea Mining and the Circular Economy: Opportunities and Challenges. International Journal of Trend in Scientific Research and Development. 2023; 2456–6470.

68. European Commission. Impacts of circular economy policies on the labour market. 2018. Available: https://circulareconomy.europa.eu/platfor
